# Co-building a patient-oriented research curriculum in Canada

**DOI:** 10.1186/s40900-019-0141-7

**Published:** 2019-02-11

**Authors:** Tim Bell, Lidewij Eva Vat, Colleen McGavin, Malori Keller, Leah Getchell, Anna Rychtera, Nicolas Fernandez

**Affiliations:** 1 Canadian Institutes of Health Research, Ottawa, ON Canada; 2 Vrije Universiteit Amsterdam, Amsterdam, the Netherlands; 3 British Columbia SUPPORT Unit, Vancouver, BC Canada; 4 Saskatchewan Centre for Patient-Oriented Research - Saskatchewan Health Quality Council, Saskatoon, SK Canada; 5 Can-SOLVE Chronic Kidney Disease Network, London, ON Canada; 6 Centre for Pedagogy Applied to the Health Sciences, Department of Family Medicine and Emergency Medicine, Faculty of Medicine, Université de Montréal, Montreal, QC Canada

**Keywords:** Patient-oriented research, Co-production/co-produced research, Patient engagement, Patient and public involvement, Training, Shared learning

## Abstract

**Plain English summary:**

*Foundations in Patient-Oriented Research* is a course designed and piloted in Canada to help patients, researchers, health care professionals and health system decision-makers gain an introductory understanding of patient-oriented research, the research enterprise, and how to work in a team. The course curriculum was co-developed by a diverse group of people with different lived experiences and relevant expertise. The course is meant to be delivered in a ‘co-learning format’ with classes comprised of all the above stakeholder groups learning together. The purpose of this study was to explore the experiences of the project leaders, developers, facilitators and patient co-facilitators who were involved in the process of co-developing, piloting and revising the curriculum.

Our findings suggest that co-developing a patient-oriented research curriculum increases its quality, uptake and credibility. The co-development process not only resulted in training that benefited the target learners, but it provided valuable learning experiences about patient-oriented research for the project leaders, developers, facilitators and patient co-facilitators. These findings and the resulting recommendations may provide guidance for other learning and development groups wishing to undertake a similar project.

**Abstract:**

**Background**

*Foundations in Patient-Oriented Research* is a course designed and piloted in Canada to build mutually beneficial relationships for conducting patient-oriented research by ensuring that relevant stakeholders – patients, researchers, health care professionals and health system decision-makers – have a common foundational understanding of patient-oriented research, the research enterprise, and team dynamics. The curriculum was co-developed by a group of patients, researchers, patient engagement experts and curriculum development experts and involved consultations with broader groups of the relevant stakeholders mentioned above. It was designed to be delivered in a ‘co-learning format’ with classes comprised of all stakeholder groups learning together. The purpose of this study was to explore the experiences of individuals involved in the process of co-developing, piloting and revising *Foundations in Patient-Oriented Research*.

**Methods**

An embedded case study was conducted with individuals who were involved in the co-development, pilot and revision of *Foundations in Patient-Oriented Research*. These individuals took on different roles during the curriculum development process, including project co-lead, developer, facilitator, and patient co-facilitator. The constant comparison method was used to inductively develop themes from the two focus group sessions.

**Results**

Discussions from the focus groups revealed the value of co-building the content, co-facilitating the course sessions, and the importance of the co-learning format. The training itself was perceived as valuable and the systematic approach to co-development was perceived as a success. Several barriers were identified, including the amount of resources, time and commitment required to complete the project. There was a notable tension between maintaining the integrity of the content and having the freedom to adapt it to local contexts. Over the course of the project, the project co-leads, developers and facilitators found that their own understanding of patient-oriented research deepened.

**Conclusions**

The findings of this study suggest that co-developing a patient-oriented research curriculum increases its quality, uptake and credibility. The co-development process not only resulted in training that benefited the target learners, but also built capacity for patient-oriented research within the project co-leads, developers, facilitators and patient co-facilitators. Our findings and recommendations may provide guidance for other learning and development groups wishing to undertake a similar project.

**Electronic supplementary material:**

The online version of this article (10.1186/s40900-019-0141-7) contains supplementary material, which is available to authorized users.

## Background

Meaningful patient and public involvement (PPI) in health research – also known in some countries as patient engagement (PE) – is not a new concept. For many decades, the traditions of participatory action research and community-based research have brought individuals and communities into active roles in the research that concerns them. More recently, governments in a number of countries have committed significant investments in co-produced health research that includes PE as an essential principle and feature [1–4]. International work and linkages across countries also continue to move forward [5]. These efforts are at the frontline of a more widespread culture shift in acceptance and understanding of PE.

As this shift continues, there is a growing need to build our collective capacity to do PE so that its true impacts continue to be captured and articulated. A key part of building this capacity is ensuring that all relevant stakeholders in the health research system – particularly, researchers, patients, health care professionals, health system decision-makers – are trained, supported, and work collaboratively towards common goals [6].

Training can be defined and offered in various ways. INVOLVE defines the term ‘training’ as “the wide range of activity that aims to help members of the public and researchers develop their knowledge, skills and experience to prepare them for public involvement in research” [7]. Training can include a variety of learning activities including group sessions with a trainer, written materials and guidance, learning-on-the-job, attending conferences, networking and shared learning with peer, online activities and university or college courses [7]. According to the 70:20:10 Model for Learning and Development, people learn 70% from ‘on-the-job training’, 20% through others (peer to peer or social learning) and 10% from formal coursework [8]. While the preferred way of learning may differ on the person, literature indicates that training in patient engagement should be co-produced with patients [7]. Furthermore, researchers and patients should learn together [7]. Co-learning can help to clarify expectations, prepare for new ways of working, help with team building and gaining a shared sense of the purpose of the involvement [7]. Members of the public and researchers at all stages of their journey with public involvement in research may benefit from training [7]. Literature shows that training provided to patients to support their active involvement in research is valuable for their own personal development, confidence, motivation and skill set, and generally has a positive impact in their lives [9]. Graduates from the European Patients’ Academy on Therapeutic Innovation (EUPATI) training course have taken on various leadership and advisory roles [10]. Benefits for researchers include, for example, gaining facilitation skills and practical knowledge on how to involve patients, such as knowledge about accessibility and compensation of patient partners [7, 11]. Training needs reported by researchers include guidance on how and when to involve patients, how to get the most out of PE, how PE benefits research, and guidance on payment [11]. Learning needs of patients include understanding how research works, including ethical considerations and how it is funded, as well as understanding the types of roles patients can have on a research team [11]. Having this knowledge provides patients with the ability and confidence to engage more directly in the research context [11].

Various organizations offer training in PE. The process of co-producing PE training programs or curricula is not frequently captured in the literature; however, there are examples that document the value-add in co-producing a training program with patients and members of the public [12, 13]. Moreover, the facilitation of PE training by patients and the public has also been demonstrated to enrich the process and outcomes [13].

The *Foundations in Patient-Oriented Research* (‘*Foundations*’) curriculum is a Canadian example of a co-produced training program. This case study explores the experiences of those who developed and piloted *Foundations* and their reflections on the project.

### The Canadian context and terminology

In 2011, the Canadian Institutes of Health Research (CIHR), the Canadian federal government’s health research funding agency, launched the Strategy for Patient-Oriented Research (SPOR) with the vision that by 2025, Canada will have demonstrably improved health outcomes and enhanced the health care experience for patients through the integration of evidence at all levels of the health care system [3]. As a national strategy, SPOR is a complex and evolving initiative that is comprised of many partners and organizations who share ownership in its implementation.

Patient-oriented research (POR) refers to “a continuum of research that engages patients as partners, focusses on patient-identified priorities and improves patient outcomes. This research, conducted by multidisciplinary teams in partnership with relevant stakeholders, aims to apply the knowledge generated to improve healthcare systems and practices” [14]. As with participatory action research [15] and community-based participatory research [16], POR possesses the same spirit of collaboration with affected individuals and communities. It also draws comparisons to what some may understand as *co-produced research* in that it involves partnerships with many relevant stakeholders and emphasizes the translation of evidence into changes in health policy and practice.

Within the context of patient-oriented research in Canada and within this paper, ‘patient engagement’ (PE) is meant as a close equivalent to the term ‘patient and public involvement’ (PPI) as defined by INVOLVE in the United Kingdom [17]. It is officially defined as the “active and meaningful collaboration of patients in governance, priority-setting, conducting research and knowledge translation.” [14]. For the purposes of this article, the term PE will be used and can be understood interchangeably with the term PPI, although it is recognized that there are a range of understandings of what the term PE means.

### The development of the *Foundations in Patient-Oriented Research* curriculum

**Fig. 1:**
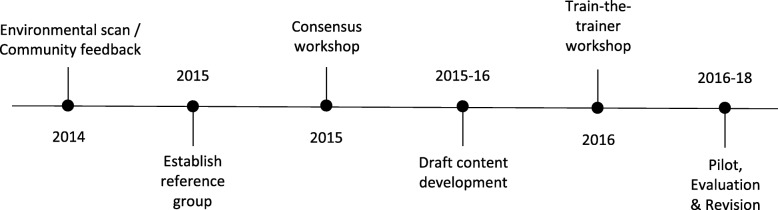
Development process of *Foundations in Patient-Oriented Research*

In the early stages of SPOR, both researcher and patient communities directly expressed a clear need for training on PE. This was a recurrent gap that was voiced at various meetings, including a national workshop to develop the SPOR Patient Engagement Framework [14]. CIHR embarked on a project to create a national curriculum on PE which could be delivered by entities funded through SPOR, such as regional POR support centres called SPOR SUPPORT Units [18] and/or pan-Canadian SPOR Networks [19]. The intent of a national-scope curriculum was to create a common, standardized set of materials and to bring alignment in understanding of PE across different organizations. The original concept intended for the materials to be generic enough so they could be further contextualized depending on the target audience. It was also assumed by CIHR that the curriculum would be only one resource in a variety of training resources and opportunities that support PE and POR. Figure 1 summarizes the development process of the curriculum.

The development process began by establishing an advisory ‘reference group’ of individuals including a number of patients, researchers, educators, a health care professional and a health system decision-maker. Members of the reference group were recruited based on existing relationships and previous involvement in SPOR, as well as the range of perspectives, knowledge and skills identified as needed for the mandate of the group. This group helped establish the initial concept for the curriculum, which was then brought to a larger consultation workshop in 2015. The 40 workshop participants – also a mix of patients, researchers, educators, health care professionals, health system decision-makers, consultants with experience in PE, and representatives from various SPOR-funded entities – validated the concept and broadened the scope beyond PE to an introduction to POR as a whole. The workshop participants also co-created an initial list of learning outcomes. One key point of divergence among workshop participants was whether the target audience of the curriculum should be exclusively patients or whether a more holistic approach should be taken and the target audience include all four stakeholder groups: patients, researchers, health care professionals and health system decision-makers. Following the workshop, the reference group supported the decision to focus on creating a ‘co-learning environment’ where individuals from all four stakeholder groups could interact and learn together. The reference group members felt there was a key opportunity for the curriculum to intentionally model the environment and interactions that a POR team would likely be facing in the ‘real world’.

At this point of the process, the reference group’s function morphed from an advisory role to a joint co-leadership role with CIHR. The learning outcomes produced from the consensus workshop were grouped into three modules: *Module 1: Introduction to Patient-Oriented Research*; *Module 2: Fundamentals of Health Research in Canada*; and *Module 3: Building Partnerships and Consolidating Teams* (more detailed information on these modules can be found in Additional file 1). The reference group members divided the modules amongst them and became the co-developers of their module’s content. The co-developers included patients, researchers and educators. Once the content was developed and tested, a national ‘train-the-trainer’ workshop was held in September 2016 to establish a country-wide cadre of certified facilitators who were tasked with piloting the draft materials over the subsequent six to nine months. At the workshop, prospective facilitators experienced the course as learners and then received training on how to deliver each module (the train-the-trainer workshop agenda can be found in Additional file 2). The outcome of the workshop was a group of 26 certified facilitators, including 12 patient co-facilitators, positioned across seven SUPPORT Units and seven SPOR Networks.

Between the period of October 2016 and August 2017, *Foundations* was piloted in whole or by single module to over 500 participants in 17 different locations spread across Canada, including urban and rural settings. *Module 1* was delivered 40 times, *Module 2* 40 times, and *Module 3* 30 times. Sessions were offered in both of Canada’s official languages – French and English. All three modules were offered five times each in French. Class sizes ranged from five to 30 participants with an average size of 14 participants. The average ratio of participants was 6:9:2:1:3 (patients/researchers/health care professionals/health system decision-makers/other perspectives), but it should be noted that the ratio of participant perspective varied greatly on a class by class basis.

An evaluation framework based on the Kirkpatrick Four-Level Training Evaluation Model [20] was employed throughout the pilot to capture various levels of feedback from participants and facilitators, which was collected centrally by CIHR. Evaluations were conducted using a series of surveys: short ‘fast feedback’ forms collected from participants immediately following each session; longer post-session surveys completed by participants online within one week of completing a session; follow-up surveys completed by participants online at least six months following the session; and facilitator feedback surveys (these surveys can be found in Additional file 3).

The pilot data revealed that 92.5% (*n* = 478/517) of participant respondents agreed or strongly agreed that the workshop materials were well-prepared and 82.6% (*n* = 427/517) of participant respondents agreed or strongly agreed that the course activities were valuable. After six months had elapsed from the time of the training, 84.6% (*n* = 44/52) of patient respondents reported that they went on to engage in POR in a variety of ways, including in governance, priority-setting, peer review, and in research teams. Both pilot facilitators and participants noted that a key strength of the curriculum was the co-learning format and the interactivity of the course. Clear feedback on how to improve the curriculum was also received for key areas of the content and its delivery, such as including more examples and practical information. Following the pilot, the developers formed ‘revision teams’ with interested pilot facilitators and used the evaluation data to make improvements to the materials. The end product of this process was a co-developed and tested national curriculum which continues to be delivered across Canada in different settings.

## Methods

The aim of this study was to explore the experiences of the development team who created the *Foundations* curriculum as well as a sample of facilitators and patient co-facilitators who piloted its initial version. The specific objectives were to:

1. Capture the experience of co-developing and co-delivering *Foundations*;
2. Explore whether the perceptions held by developers and facilitators about patient-oriented research (POR) changed throughout the process and, if so, how; and,
3. To make recommendations to enhance training for patients and researchers for Canadian and international learning and development groups.

### Study design

In order to achieve the above objectives, a case study approach with embedded levels was selected [21]. This allowed the observation of the study object (the process of co-developing a curriculum) through exchanges between study participants following the completion of the pilot. A participatory research approach was taken in which research participants were also offered the opportunity to become involved in various phases of the research project as co-investigators. All participants agreed to be part of the investigator team.

### Sample and recruitment

Participants were recruited by the project lead. To ensure a variety of perspectives, a mix of developers, facilitators and patient co-facilitators were invited based on geography (West, Central and East Canada) as well as affiliation with the development team. Patient co-facilitators were invited by their local facilitator via email or phone if not already involved in the development team. In total, 7 participants agreed to take part in the study. Some participants played multiple roles during the development and pilot process, as indicated in Table 1. The roles of project co-lead, developer, facilitator, and patient co-facilitator are described below Table 1.

**Table 1:** Focus group participant roles in the pilot of the *Foundations* curriculum

Participant	Project Co-lead^a^	Developer^b^	Facilitator^c^	Patient Co-facilitator^d^
A	X	X	X	
B	X	X	X	
C	X	X	X	
D	X	X		
E			X	
F				X
G				X

^a^Project Co-lead: Individuals who led and made joint decisions about the curriculum development process, train-the-trainer session, pilot, revisions, as well as input into the roll-out of the finalized version

^b^Developer: Individuals with education and facilitation background who led the development of the curriculum content and supporting materials, such as a facilitator guide

^c^Facilitator: Individuals selected from regional SPOR SUPPORT Units or SPOR Networks who delivered the curriculum on multiple occasions during the pilot period

^d^Patient Co-facilitator: Individuals with lived experience of a health issue, engaged by a SPOR SUPPORT Unit or SPOR Network, who co-delivered the curriculum with another facilitator

### Data collection

The case study consisted of two semi-structured, two-hour focus group sessions involving all seven participants. These sessions were conducted and recorded using online meeting technology and both were led by a professional facilitator.

The purpose of the first focus group session was to capture the experiences of the participants in their various roles in the development and piloting process. Prior to this session, the group created a set of open-ended questions to prompt discussion and draw out the personal experiences of each group member. Following the first focus group session, a web link of the recording was shared amongst the participants.

In advance of the second focus group session, participants listened to the first focus group recording and took note of anything that was particularly interesting or significant to them. Beyond this exercise, the first focus group session recording was not analyzed further. The purpose of the second focus group session was to discuss the highlights brought forward by each participant and to begin identifying recommendations for training groups. The recording of this session was then transcribed.

### Data analysis

The constant comparison method was used to analyze and inductively derive themes from the transcript [22]. A coding template was developed by two investigators (TB, LV). The template included participant number, role, raw data (quote), code, sub-theme and theme. Independent from one another, two investigators (TB, LV) broke the transcript down into discrete pieces or thoughts, performed an open coding of these pieces, and then identified both themes and sub-themes from the resulting codes. Both investigators (TB, LV) then compared their coding and thematic analyses for agreement or, where there was divergence, to choose a consensus code or theme. The two sets of themes were therefore consolidated into a single one. This consolidated set was shared with the rest of the investigators electronically for one further refinement and validation.

### Reporting

Two investigators (TB, LV) created a first draft manuscript based on the analysis. All investigators reviewed the draft and made edits. A final manuscript was created by two investigators (TB, LV) and reviewed and approved by all investigators. Note that all participants were also investigators in this study and are listed as co-authors of this paper. We referred to the GRIPP 2 reporting template to ensure this manuscript reports relevant information regarding PE/PPI (see Additional file 4).

## Results

The thematic analysis of the second focus group revealed nine themes about the development and piloting process (Table 2). Each key theme also included a number of sub-themes or topics. Results can be linked to various phases of the development and pilot process.

**Table 2:** Themes and sub-themes about the *Foundations in Patient-Oriented Research* development and pilot process derived from the second focus group session

Theme: The value of co-building the curriculum Participants indicated that a critical aspect of the project was bringing relevant stakeholders together to make decisions about the scope, format and content of the curriculum.	
Sub-theme: Drawing on expertise from all stakeholders	Quote: “I would’ve never been able to create this content by myself.” *[Participant B, Project Co-lead/Developer/Facilitator]* Quote: “Without that team approach where we all brought our expertise, I don’t think we would’ve had training in many of the provinces.” *[Participant A, Project Co-lead/Developer/Facilitator]* Quote: “I also think that really helped with the standardization of language.” *[Participant B, Project Co-lead/Developer/Facilitator]*
Sub-theme: Having shared experiences	Quote: “It was great to be around like-minded people… it helped me feel confident in delivering the training after.” *[Participant E, Facilitator]*
Sub-theme: Having learning experiences	Quote: “The co-learning I think not only applied during the workshops when we were facilitating them with the patients and researchers and other stakeholders, but I think we as a development team, we were learning as we went along.” *[Participant C, Project Co-lead/Developer/Facilitator]*
Sub-theme: Building relationships	Quote: “By just going on the [central website containing the list of sessions] or by contacting [a project co-lead], those collaborations occurred, which I think is a key piece that we need to continue – that infrastructure is still there and people in their local area.” *[Participant B, Project co-lead/Developer/Facilitator]*
Theme: The value of the co-learning model Participants indicated that the most important feature of the curriculum was the co-learning model, i.e., having relevant stakeholders learning and interacting in the same space together.	
Sub-theme: Having shared experiences	Quote: “Getting people … in a room together, learning face-to-face and the power of that in terms of breaking down barriers, tackling the issue of power imbalances that can exist on a research team.” *[Participant C, Project Co-lead/Developer/Facilitator]*
Sub-theme: The usefulness of story-telling	Quote: “What I found was really helpful and was the balance between partially lecturing but especially the stories and the examples because they bring it across. When we think of what we remember later on, we picked up a few key notes maybe from a lecture, but stories, examples, especially presented by different people, that really sticks in the memory.” *[Participant B, Project Co-lead/Developer/Facilitator]*
Sub-theme: Catalyzing relationships to conduct POR	Quote: “Co-learning is the intervention that builds teams.” *[Participant C, Project Co-lead/Developer/Facilitator]* Quote: “You could almost physically see people relaxing into themselves and the relationships … that came out of the co-learning format.” *[Participant F, Patient co-facilitator]*
Theme: The value of co-facilitating the course Participants indicated that co-facilitating the sessions together with a patient or with someone with relevant expertise (e.g., health research landscape, patient engagement) was valuable to imparting the content effectively.	
Sub-theme: Patients feel like a true partner by co-facilitating	Quote: “Being taken serious and being listened to is what I mean by moving past tokenism and being, really, a full member of the team.” *[Participant G, Patient co-facilitator]*
Sub-theme: Building relationships	Quote: “…not just in the facilitation, but then discussions around the course afterwards and exchanges [about the] experience, the interaction in-between different patient partners.” *[Participant G, Patient co-facilitator]*
Theme: Experienced enablers or strengths of the process Participants identified a number of factors that positively influenced the development process and/or the pilot.	
Sub-theme: Co-building as a result of the initial workshop	Quote: “I think a really great example of [the co-building process] was the initial workshop that brought people together to talk about what should be covered and what emerged from that workshop around the importance of this idea of co-building and co-learning.” *[Participant C, Project Co-lead/Developer/Facilitator]*
Sub-theme: Having common goals and/or personal motivation	Quote: “People on this group started working out of personal interest, not because we were dictated to. So we came to the table out of desire and interest in patient engagement, not as a ‘thou shalt’. And I thought that that really may have had an impact on how well the team worked together as well as how the modules were created.” *[Participant B, Project co-lead/Developer/Facilitator]*
Sub-theme: Adaptability/flexibility of the delivery	Quote: “I loved that we were allowed to do that in that we didn’t require teams to come to the training, it was just open to anyone who was engaged in POR or wanting to be engaged. And you didn’t have to be attached to a team, but yet if you were we strongly encouraged it.” *[Participant B, Project Co-lead/Developer/Facilitator]* Quote: “I think different people mentioned the desirability of customizing the curriculum for local delivering, like being able to give local examples… just facilitator preferences. I know, for example, for Module 2 I had different preferences around something as simple as slide transitions and that was kind of again butting up against the need to maintain integrity of the curriculum and also, you know, locking it down for the pilot.” *[Participant C, Project Co-lead/Developer/Facilitator]* Quote: “I really liked as well that we were able to pilot via video conferencing with one of our rural areas.” *[Participant E, Facilitator]*
Sub-theme: Co-facilitation with people with lived experience	Quote: “I really felt as a partner in the co-facilitation. I felt heard and valued in terms of discussions.” *[Participant G, Patient co-facilitator]*
Sub-theme: Validating the course content through evaluations and feedback	Quote: “The pilot collected the perspectives of the different learners, and I think that was really important to [incorporate this].” *[Participant D, Project co-lead/Developer]* Quote: “[The] evaluation framework that we used during the pilot process [was] exactly what we built the revisions upon.” *[Participant D, Project co-lead/Developer]* Quote: “The feedback we got through the pilot – we can actually use some of the feedback almost as marketing when advertising the curriculum.” *[Participant C, Project co-lead/Developer/Facilitator]*
Sub-theme: Credibility of the systematic development process	Quote: “I liked the reference group idea … although … [it] morphed or changed over time, I still think it was a great way to … credibly build a project together across the country.” *[Participant D, Project Co-lead/Developer]* Quote (about the train-the-trainer workshop): “It was great to be around like-minded people … it helped me feel confident in delivering the training after.” *[Participant E, Facilitator]* Quote: “I think we might’ve seen in the past and in other areas of our work where something’s evolved and that’s just handed to the world as-is, but this was a very well-thought out way of really, piloting it.” *[Participant F, Patient co-facilitator]* Quote: “I think the pilot was really necessary for two reasons. One: for improvement, and the other one for credibility.” *[Participant C, Project Co-lead/Developer/Facilitator]*
Theme: Beliefs about the success of the process Participants brought forward their perceptions about what the curriculum achieved and how the curriculum development and pilot process was successful to them.	
Sub-theme: Improved understanding of POR	Quote: “The knowledge [exchange] that has happened … testified to the changes in what we thought and what we saw in the people around us.” *[Participant A, Project co-lead/Developer/Facilitator]* Quote: “There’s [anecdotal evidence] suggesting that groups and teams who took the course actually fared better than those who did not when it came to getting funding or when it came to their [success] down the line.” *[Participant D, Project Co-lead/Developer]*
Sub-theme: Widespread uptake of the curriculum	Quote: “I think the impact of the training has been wide-spread… On one hand, you have more people who are knowledgeable of patient-oriented research and on the other hand you also see groups developing further [training].” *[Participant A, Project Co-lead/Developer/Facilitator]* Quote: “We achieved a national resonance with this foundational curriculum and that’s not negligible.” *[Participant A, Project Co-lead/Developer/Facilitator]*
Theme: Understanding POR Participants noted observations about how they and the course learners changed in their understanding POR.	
Sub-theme: Understanding patient engagement within the context of POR	Quote: “What I found just super rewarding is working with the participants and to see that they start to see the value of patient engagement, especially with the exercise with the research cycle and starting to understand where patient engagement fits in.” *[Participant E, Facilitator]*
Sub-theme: Researchers/patients skills in PE	Quote: “It struck me how much this is new for the researchers. And that’s because researchers are trained to do research in a certain way, especially in health sciences, and, you know, participatory action research is not among the tools in their toolbox.” *[Participant A, Project Co-lead/Developer/Facilitator]* Quote: “It’s not just telling a story; it’s also analyzing it and being part researching themselves in a way. [Patients are] inventorying what they’ve learned from experience, and also finding ways to articulate it and finding ways to make it meaningful in the exchanges and the decisions that are made in a research team.” *[Participant A, Project Co-lead/Developer/Facilitator]*
Sub-theme: Evolving concept, knowledge and skills	Quote: “We’ve all deepened our knowledge of what POR is, should be, could be, etc.” *[Participant A, Project co-lead/Developer/Facilitator]* Quote: “Another example of our evolution of understanding was, it started with that initial workshop back ... when we decided that the curriculum needed to be about more than just patient engagement. That patient-oriented oriented research, yes patient engagement is foundational to that, but, the emphasis on the multi-disciplinarity idea and the bringing together of all the stakeholder different perspectives in order to do ‘patient-oriented’.” *[Participant C, Project Co-lead/Developer/Facilitator]* Quote: “I knew about patient engagement, but I really didn’t know that much about patient-oriented research. But by being a part of this, I was able to tap into experts in the development of the training … [that] was really key, because they were able to share for Module 1 and Module 2 what was the health research side, broader than just patient engagement, and I felt like that for me enabled me to actually deliver the content.” *[Participant B, Project Co-lead/Developer/Facilitator]*
Theme: Barriers or tensions experienced during the process Participants identified a number of factors that impeded or created challenges during development and/or the pilot process.	
Sub-theme: Coordination	Quote: “Just the level of planning and team building and check-ins that were required for facilitating and co-facilitating, and supporting the patient co-facilitators.” *[Participant E, Facilitator]* Quote: “That is one of the characteristics of co-development; you have to build in affordances for unseen things, unpredictable things.” *[Participant A, Project Co-lead/Developer/Facilitator]* Quote: “The execution of it required a lot of follow-up … to get people to share … the planned sessions that they had.” *[Participant D, Project Co-lead/Developer]* Quote: “The [challenge] was more synchronizing all the different SUPPORT Units and SPOR Networks.” *[Participant A, Project Co-lead/Developer/Facilitator]*
Sub-theme: Resources	Quote: “I had some frustrations around the amount of time it took us to accomplish what we’ve accomplished, which has been a lot, but it’s also taken us three years to get here.” *[Participant C, Project Co-lead/Developer/Facilitator]* Quote: “So I would argue that a lot of resources [needed to complete the project] have been volunteer.” *[Participant C, Project Co-lead/Developer/Facilitator]* Quote: “I was thinking about how to … shorten the timeframe for the project and I … think more protected time to do something like this is necessary.” *[Participant D, Project Co-lead/Developer]* “Going forward for sustainability you need to have resources put in place to have the continuous refreshing and coordination of something like this.” *[Participant D, Project Co-lead/Developer]*
Sub-theme: Commitment	Quote: “I would say my experience as a train-the-trainer leader, the people who had passion actively engaged in the train-the-trainer and there were a few people who were told to attend and they didn’t participate actively.” *[Participant B, Project Co-lead/Developer/Facilitator]* Quote: “I don’t actually think that it was more resources that were necessarily needed as much as we needed [more] leadership commitment [to make the project go faster].” *[Participant B, Project Co-lead/Developer/Facilitator]*
Sub-theme: Communication	Quote: “There could’ve been a bit clearer, upfront statements on a website to talk about the curriculum and … have some clear messaging about what it is and what stage it’s at.” *[Participant D, Project Co-lead/Developer]*
Sub-theme: Tension between the freedom to adapt content versus maintaining its integrity and core messages	Quote: “That was one of the things in the train-the-trainer workshop we … outlined; what was fixed and what was flexible … people knew that they had some ability to manipulate the delivery of it.” *[Participant B, Project co-lead/Developer/Facilitator]* Quote: “Any frustrations that I’ve felt with the rigidity of slides, and this or that, I understood from a science research perspective that there needed to be somewhat of a consistent delivery across the country so that we could evaluate it and enhance it that way.” *[Participant F, Patient co-facilitator]* Quote: “In general, people adapted the time, they didn’t minimize it to the extreme.” *[Participant A, Project co-Lead/Developer/Facilitator]*
Theme: Environmental context Participants discussed certain the underlying context in which the project was executed.	
Sub-theme: Existing beliefs about POR	Quote: “We’re up against people who don’t really value POR. I mean, if they’re not ready to meet as a team and go through training together, it says something about what they really think about POR.” *[Participant A, Co-lead/Developer/Facilitator]*
Sub-theme: Existing infrastructure	Quote: “I think having the initial infrastructure of the SUPPORT Units and having the research Networks in SPOR were critical to getting something as far reaching.”*[Participant D, Co-Lead/Developer)*

### Design and content development phase

The value of co-building the curriculum was widely emphasized. Focus group participants reported that the variety of expertise was key in making decisions about the scope, format and content of the curriculum. The initial workshop that brought people together to talk about the content and what emerged from that workshop around the importance of co-building and co-learning was seen as an important step in the development process. The resulting change in trajectory due to the co-building process was also something that could not be predicted at the very beginning of the project. One participant stated:

*“I think this is a clear example of co-building and we should be proud on that. What it means is that there is no predefined outcome. I think that is a key thing.” [Participant A, Project Co-lead/Developer/Facilitator]*

Furthermore, participants reported the value of having a constant feedback loop across the development team while everyone worked independently on their pieces of the content. The development process itself was received as a co-learning journey.

### Train-the-trainer and piloting phase

Several focus group participants commented positively on the usefulness of the train-the-trainer workshop. Some expressed that it was motivating to be with like-minded people and reported that they felt comfortable delivering the sessions after the train-the-trainer workshop. One patient co-facilitator mentioned that she felt a little uncomfortable delivering *Module 2: Fundamentals of Health Research in Canada* without having a background in health research. The pilot was seen as key for improvement of the curriculum and for credibility. Including the SPOR SUPPORT Units and Networks was seen as critical to enabling delivery at 17 different locations across the country with over 500 participants over nine months. The national coordination provided by CIHR was seen as critical to convening these groups together, providing updates during the pilot, and supporting connections between facilitators wherever possible. There were varied opinions on the value of having such a large number of facilitators and groups responsible for delivering the pilot, which was seen as beneficial for reaching more audiences but was also seen as lengthening and adding complexity to the process. All focus group participants also stated the co-learning environment was a key success factor. Facilitators said it was powerful to have all the different stakeholders in the room as they were able to see the perceived barriers between groups (such as patients and researchers) begin to fade. Delivering the curriculum with a patient co-facilitator was also seen as valuable. The combination of theory, personal stories and group discussions was well-received. One facilitator mentioned:

*“The balance between partially lecturing [and] the stories and the examples was great, because they bring [the concepts] across. When we think of what we remember later on, we pick up a few key notes maybe from a lecture, but stories [and] examples, especially presented by different people, that really sticks in the memory.” [Participant B, Project Co-lead/Developer/Facilitator]*

A patient co-facilitator who was also able to share personal stories during the workshops said:

*“I really felt as a partner in the co-facilitation. I felt heard and valued in terms of the discussions. Not just in the facilitation, but then in the discussions around the course afterwards and exchanges [about the] experience.” [Participant G, Patient co-facilitator]*

Regarding the question of whether to allow changes to the curriculum, comments spoke to both content and delivery methods. During the pilot, the content was locked and a few facilitators mentioned that they would have liked to have had the opportunity to adjust it more to their local context. Others felt that it was important not to allow changes during the pilot so as to maintain the functionality of the evaluation framework and the integrity of the key messages and concepts. Facilitators appreciated flexibility in terms of delivery; for example, having the ability to deliver only segments; using technology to deliver the modules in real-time at different (rural) locations; and delivering the modules to entire research teams or programs of research versus offering sessions openly to anyone who was interested. One facilitator shared:

*“[We were] able to pilot having the modules broken up into smaller segments, which worked really well for people who were dealing with current medical issues, they couldn’t sit through a whole eight-hour day of training, so we offered it out in smaller segments in two- to three-hour sessions instead and that was really well-received.” [Participant C, Project Co-lead/Developer/Facilitator]*

### Evaluation and revision phase

The evaluation framework was seen as a part of a larger systematic process which lent credibility to the curriculum. Participants felt the evaluation was successful in collecting feedback from all stakeholder groups in order to inform the revisions. Participants also reported that the evaluation helped to create an open atmosphere. One person mentioned:

*“I really appreciated that we didn’t say we knew everything and that this was the gospel, forever and ever amen. Instead, we said ‘this is what we know for now about patient-oriented research and we’re going to continue to evolve and grow the content’.” [Participant B, Project Co-lead/Developer/Facilitator]*

Participants made a number of initial recommendations for other development groups wishing to undertake a similar project:

- Have all key stakeholders – including patients, researchers, health care professionals and health system decision-makers – at the table from the beginning;
- Do not underestimate the time and resources required to complete the project, ensuring contingencies are built into the project plan;
- If multiple organizations are involved, ensure clear roles and relationships are articulated and committed to by the leadership and staff involved;
- Personal interest in patient engagement and the motivation to be part of the endeavor is a key to success;
- Ensure a training coordinator role exists to manage communications, maintain a national listing of sessions offered and facilitate a community of practice across the country for facilitators and co-facilitators.

## Discussion

The findings of this case study describe the value proposition of co-building a curriculum, from early conceptualization to the end stages of evaluation and revision. The trajectory of the development process was greatly influenced by the advice provided or decisions made by groups comprised of patients, researchers, health care professionals and health system decision-makers. The co-building process was viewed as critical to achieving the end product and the ability to pilot it nationally in different contexts. The co-building approach also lent credibility to the process and content.

Despite these perceived benefits, a co-developed curriculum does not necessarily mean a one-size-fits-all training solution will be produced. A key assumption about the curriculum before its conceptualization began was that it would exist as one resource in a myriad of different training opportunities that support PE specifically and POR more broadly. It might be that alternative types of education such as ‘learning on the job’ and coaching are more suited or a combination of training opportunities as also suggested by Dudley et al. (2015) [10] and the 70:20:10 Framework [7]. While the content of *Foundations* is meant to be national in nature and culturally neutral, additional effort is needed on behalf of facilitators to tailor it to local cultures, communities or populations. Other research also suggests that those providing training and wishing to improve its uptake need to articulate what both researchers and patients can expect to gain from training [10].

The co-building process also introduced a greater degree of complexity to the project due to the number of people involved and the geographical spread between them, having to align schedules, and having to navigate various perspectives when making decisions. This protracted the overall timeline and increased the amount of financial and human resources needed, the latter of which was sometimes volunteer in nature. As such, for a national-scale, co-development approach, it is recommended that there is strong buy-in and commitment from leadership in place when embarking on such an endeavor, as well as thorough coordination.

*Foundations* offered both traditional course work as well as peer to peer learning activities. We found that the most crucial aspect of the content design, however, was the co-learning approach. Since POR or co-produced research involves relevant stakeholders working together, it was imperative that this principle be modeled in the course format itself. Our results support the findings of Horobin et al. (2017) who also found that interaction between participants – the sharing of varied experiences and knowledge – and a ‘learn by doing’ approach was of particular value [11]. The project co-leads, developers and facilitators also experienced shared learning by undertaking the co-building approach. By developing content and piloting it, their own understanding of POR was deepened.

One aspiration of implementing *Foundations* was that the course would dovetail into further training or real-world POR opportunities offered through SPOR SUPPORT Units or SPOR Networks. While this was not a requirement or goal of the pilot, some SUPPORT Units and Networks have gone on to integrate *Foundations* into their overall slate of activities. For example, some have built further modules and tools as a continuation of the content. Some learners who completed *Foundations* have started their own POR projects while others became part of an existing POR project as a partner. Furthermore, some patient learners became involved within SUPPORT Units or Networks as a patient advisory council member. The scope and extent to which these outcomes have occurred has not yet been measured.

### Strengths and limitations

Little research specifically describes the development of training programs in POR and the roles that patients and other stakeholders can have in this process. This study provides valuable guidance for learning and development groups who may wish to create similar training programs. The seven investigators who conducted this study were also the study participants. All investigators participated in the case study design, data analysis, data interpretation and manuscript production. This can be seen as a strength of the study and is in line with the co-developing and co-learning approach of this initiative.

Only a sample of all developers, facilitators or patient co-facilitators participated in the focus group sessions in this case study, which can be seen as a limitation. It is reasonable to assume that the experiences of the other developers, facilitators or patient co-facilitators who did not participate could differ from those reported in this paper. Our study was not intended to formally assess each stakeholder’s perspective and the impact that being part of this initiative had on patients and other stakeholders. Such assessment would potentially identify more in-depth information and recommendations. While the perceptions of developers and facilitators are important to inform future training programs, the experience of participants are as important to learn about the effectiveness and value of training programs. A separate formal evaluation study was done to inform the content revisions of the curriculum. The facilitators who participated in this case study gave some reflections on what they have heard and learned from participants.

## Conclusions

The perspectives brought forward from this study suggest that the *Foundations* development process has demonstrated the feasibility and added value of co-developing and piloting a curriculum in PE and POR on a national level. The findings of this case study suggest that co-developing a POR curriculum with patients, researchers, health care professionals, health system decision-makers and other relevant stakeholders (i.e., educators) increases its quality, uptake and credibility. The co-development process not only resulted in training that provided the intended audiences with a valuable learning opportunity, but it also built capacity for POR within the project co-leads, developers, facilitators and patient co-facilitators. The *Foundations* curriculum has been modified based on the feedback from curriculum participants and facilitators. The co-development approach and co-learning delivery format has been maintained. The revised version of *Foundations* is currently being used by various SUPPORT Units and Networks to further train relevant stakeholders. In the future, additional revisions might be made as POR is still in development; new evidence on ‘how to do POR’ and ‘the value of POR’ can further inform training programs. Furthermore, training needs and preferences may change over time. More research is needed to better understand the long-term impacts of training programs and to identify the additional learning needs of all stakeholders.

## Additional files

Additional file 1: Overview of *Foundations in Patient-Oriented Research* and modules. (PDF 562 kb)

Additional file 2: *Foundations in Patient-Oriented Research* – Train-the-trainer Workshop Agenda. (PDF 630 kb):

Additional file 3: *Foundations in Patient-Oriented Research* – Overview of In-session and Post-session Evaluation Elements. (PDF 906 kb)

Additional file 4: Completed GRIPP-2 Long Form. (PDF 366 kb)

## Abbreviations

CIHR: Canadian Institutes of Health Research
Foundations: Foundations in Patient-Oriented Research
PE: Patient Engagement
POR: Patient-Oriented Research
PPI: Patient and Public Involvement
SPOR: Strategy for Patient-Oriented Research
