# Supplementary material for: Co-building a patient-oriented research curriculum in Canada
Source: Res Involv Engagem. 2019 Feb 11;5:7. doi: 10.1186/s40900-019-0141-7 (PMC6369555; doi:10.1186/s40900-019-0141-7)
Supplement: Supplementary file 1 — Overview of Foundations in Patient-Oriented Research and modules. (PDF 562 kb) [file 40900_2019_141_MOESM1_ESM.pdf]

Additional file 1: Overview of *Foundations in Patient-Oriented Research* and modules

Course description

This highly interactive course is designed to build mutually beneficial relationships for conducting patient-oriented research by ensuring that all stakeholders – patients, researchers, health care professionals and health system decision-makers - have a common foundational understanding of patient-oriented research, the research enterprise, and team dynamics. The course is designed to be delivered in a co-learning format with classes comprised of all stakeholder groups, learning together. It emphasizes group discussions that enable participants to appreciate diverse perspectives and to begin to develop relationships that may lead to successful research teams.

Module 1: Patient-Oriented Research

The goal of this module is to foster an understanding of patient-oriented research and provide an opportunity for people to assess how they may wish to become engaged. This module is suitable for patients, researchers and other stakeholders – including health care professionals and health system decision-makers – who want to know more about patient-oriented research.

Learning outcomes

By the end of this module, participants should be able to:

1. Define patient-oriented research and describe how it is different from more traditional health research
2. Articulate the benefits and challenges of involving patients in health research
3. Appreciate the various roles that patients can meaningfully and actively play in health research, including governance, priority setting, the conduct of research and knowledge translation
4. Identify the kinds of roles patient partners are interested in
5. Assess the unique strengths that patients may bring, not only as patients but through their other personal, educational and professional experiences
6. Describe the various levels of engagement as outlined by the International Association of Public Participation (IAP2)
7. Appreciate the guiding principles that underpin patient engagement in health research: inclusiveness, support, mutual respect and co-building.
8. Describe examples of ways patients have been involved in patient-oriented research
9. Outline the practical considerations for engaging patients as partners in health research – e.g., developing relationships with communities and individuals, defining roles and responsibilities, compensation, creating culturally and socially safe environments
10. Compare patient-reported outcome measures and patient-reported experience measures with measures traditionally used in health research

11. Appreciate the value of personal stories and how they contribute to a better understanding of the needs, values and preferences of patients
12. Identify future learning needs

Module 2: Fundamentals of Health Research in Canada

The goal of this module is to engage patients, other non-researchers (e.g., health care professionals and system decision-makers) and researchers in conversations about what health research is. This overview will enable learners to situate their interests and roles within a well-rounded understanding of research processes.

Learning outcomes

By the end of this module, participants should be able to:

1. Describe the purpose of health research
2. Describe who typically conducts health research studies and the traditional role of patients as study subjects
3. Describe the role of the Canadian Institutes of Health Research (CIHR) and other health research funders
4. Describe the diversity of health research topics and studies
5. Participants will be aware that there are different research designs and methodologies
6. Define knowledge translation/knowledge exchange
7. Describe the characteristics of a good research question using the FINER acronym (feasible, interesting, novel, ethical and relevant)
8. Describe the stages of a research study
9. Describe the peer review process
10. Describe the ethical considerations for health research and how ethical practices are assured

Module 3: Building Partnerships and Consolidating Teams

The goal of the module is to learn how to support a research team as it goes through stages of team development.

Learning outcomes

The module is intended to enable participants to:

1. Learn about Tuckman's model of team development.

2. Learn tools to support the team as it goes through stages of development.
3. Learn to apply this information to your research team.
