# Supplementary material for: Co-building a patient-oriented research curriculum in Canada
Source: Res Involv Engagem. 2019 Feb 11;5:7. doi: 10.1186/s40900-019-0141-7 (PMC6369555; doi:10.1186/s40900-019-0141-7)
Supplement: Supplementary file 2 — Foundations in Patient-Oriented Research – Train-the-trainer Workshop Agenda. (PDF 630 kb): [file 40900_2019_141_MOESM2_ESM.pdf]

Additional file 2 – Train-the-trainer Workshop Agenda

Foundations in Patient-Oriented Research

Curriculum Train-the-Trainer Workshop

OVERALL WORKSHOP GOAL | To develop a cadre of trained facilitators from SPOR SUPPORT Units and SPOR Networks who will to pilot the *Curriculum* within their jurisdictions.

OBJECTIVES | To provide trainers an overview of the initiative and why it is being developed;
To let trainers participate as learners and experience the pilot *Curriculum* first-hand;
To give trainers an opportunity to practice facilitating parts of the pilot materials; and,
To provide an overview of the evaluation framework for the pilot materials

ASSUMPTIONS | *Foundations in Patient-Oriented Research* is situated within a broader system of learning and support that helps build capacity for patient-oriented research.

Participants attending this train-the-trainer workshop meet the essential requirements provided to the SUPPORT Units and Networks and will deliver the Curriculum – *i.e., the materials in their current pilot form* – at least twice by the end of March 2017, using the evaluation framework that has been created.

DOCUMENTATION | Beyond this agenda and an overview slide deck, *Curriculum* materials (including slides, facilitator guides and participant handbooks) will be provided to participants at the time of the workshop.

GENERAL OUTLINE | Day 1**:** Welcome, Context-setting and Module 1
Day 2**:** Module 2 and Practice Sessions for Modules 1 and 2
Day 3**:** Module 3
Day 4**:** Practice Session for Module 3, Evaluation Framework and Wrap-up

Day 1 | Tuesday, September 13th, 2016<br><br>8:30 am – 5:00 pm		
**Objectives**<br><br>• To provide an overview of the *Curriculum* project and pilot phase<br>• For participants to experience **Module 1** as learners		
**Time**	**Activity**	**Notes**
8:00 – 8:30 am	**Registration**	
8:30 – 8:45 am	**Welcome and introductions**<br><br>• Opening remarks<br>• Introduction of facilitators, Reference Group members	
8:45 – 9:15 am	**Overview of the train-the-trainer workshop**<br><br>• Overview of workshop<br>• Housekeeping, expectations and ground rules<br>• Introduction of evaluation framework	
9:15 am – 5:00 pm	**Module 1: Patient-oriented research**	*Session includes two 15-min breaks (one a.m., one p.m.) and a 45-min lunch at approx. 12:00 pm*
5:00 pm	**Wrap-up of Day 1**<br><br>• Collect any quick reflections<br>• Review Day 2 agenda	
**Day 2 | Wednesday, September 14th, 2016**<br><br>**8:30 am – 5:00 pm**		
**Objectives**<br><br>• For participants to experience **Module 2** as learners<br>• For participants to practice facilitating parts of **Modules 1 & 2**		
**Time**	**Activity**	**Notes**
8:00 – 8:30 am	**Networking time** (optional)	

8:30 – 10:45 am	**Module 2: Fundamentals of health research in Canada**	
10:45 – 11:00 am	**Break**	
11:00 am – 5:00 pm	**Module 1 and 2 practice sessions**	*Includes a 45-min lunch at approx. 12:00 pm and a 15-min break in the afternoon*
5:00 pm	**Wrap-up of Day 2**<br>• Collect any quick reflections<br>• Review Day 3 agenda	
**Day 3 | Thursday, September 15th, 2016**<br>**8:30 am – 4:00 pm**		
**Objectives**<br>• For participants to experience **Module 3** as learners		
**Time**	**Activity**	**Notes**
8:00 – 8:30 am	**Networking time** (optional)	
8:30 – 4:00 pm	**Module 3: Building partnerships and consolidating teams**	*Session includes two 15-min breaks (one a.m., one p.m.) and a 45-min lunch at approx. 12:00 pm*
4:00 pm	**Wrap-up of Day 3**<br>• Collect any quick reflections<br>• Review Day 4 agenda	
**Day 4 | Friday, September 16th, 2016**<br>**9:00 am – 3:00 pm**		
**Objectives**<br>For participants to practice facilitating parts of **Module 3**<br>• To provide participants with an overview of the evaluation framework and tools<br>• To provide participants with a Q & A session with facilitators		

Time	Activity	Notes
8:30 – 9:00 am	**Networking time** (optional)	
9:00 am – 12:15 pm	**Module 3 practice session**	*Session includes a 15-min break*
12:00 – 1:00 pm	**Lunch**	
1:00 – 1:30 pm	**Overview of evaluation framework**<br>• Evaluation framework principles and components<br>• Review of the standard of procedure for facilitators	
1:30 – 1:45 pm	**Break**	
1:45 – 2:45 pm	**Questions & Answers**	
2:45 – 3:00 pm	**Concluding remarks**	
