# Supplementary material for: Co-building a patient-oriented research curriculum in Canada
Source: Res Involv Engagem. 2019 Feb 11;5:7. doi: 10.1186/s40900-019-0141-7 (PMC6369555; doi:10.1186/s40900-019-0141-7)
Supplement: Supplementary file 3 — Foundations in Patient-Oriented Research – Overview of In-session and Post-session Evaluation Elements. (PDF 906 kb) [file 40900_2019_141_MOESM3_ESM.pdf]

Overview of In-Session and Post-Session Evaluation Elements

Each Module will be evaluated during the pilot phase using the following elements:

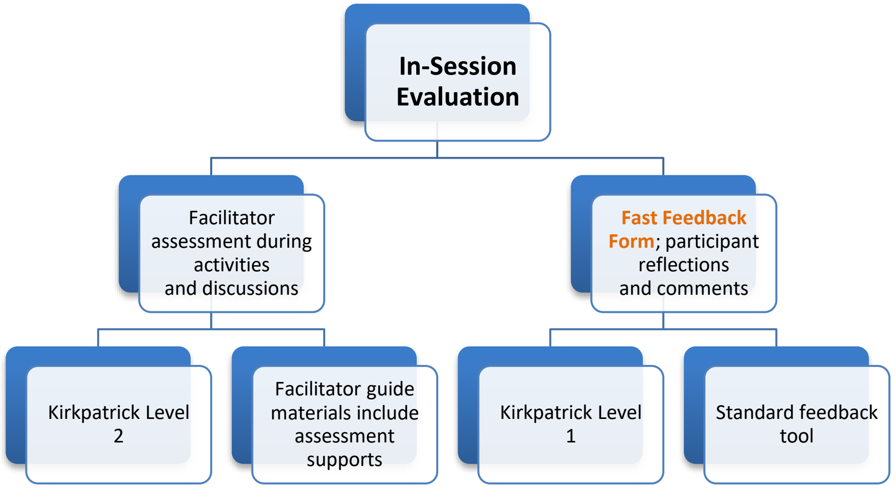

**Level 1:** REACTION	*How did learners feel about the learning?*
**Level 2:** LEARNING	*Did they learn what we intended?*
**Level 3:** BEHAVIOUR	*Has what they learned changed their actions?*
**Level 4:** RESULTS	*Has the learning changed*

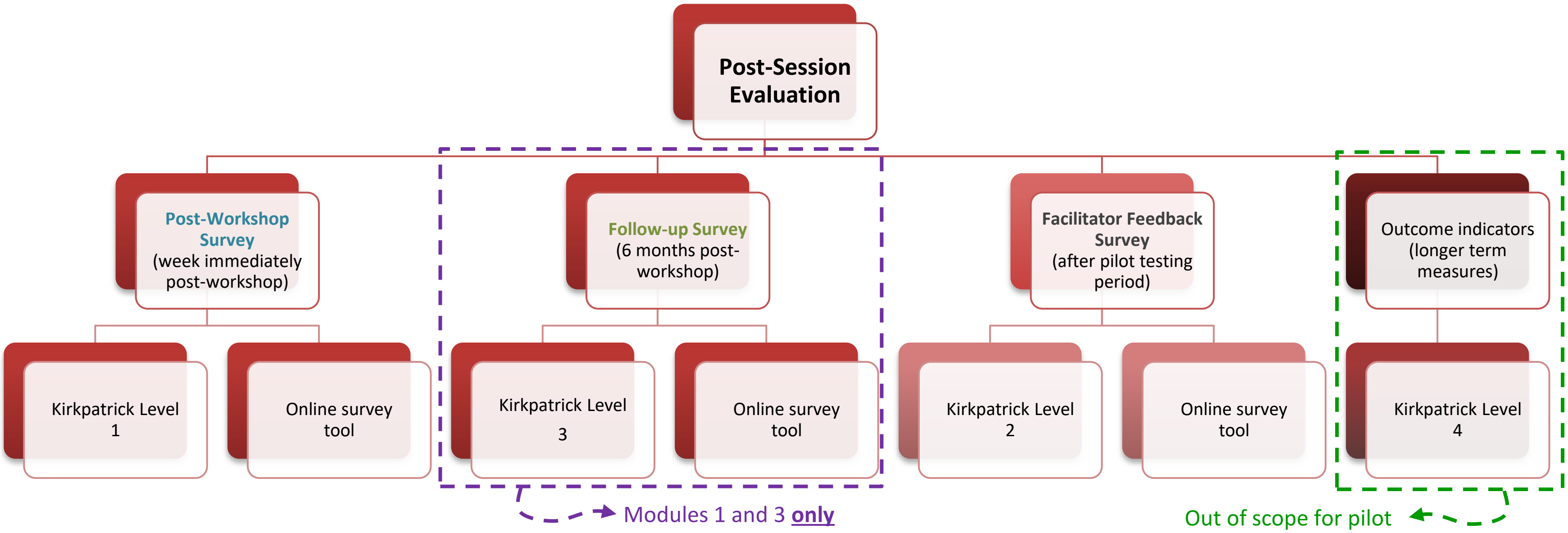

Module 1: Patient-Oriented Research

Key Evaluation Elements

Learning Outcomes	In-Workshop Evaluation Elements	Post-Workshop Evaluation Elements
A. Shared meaning and common language		
Define patient-oriented research and describe how it is different from more traditional health research.	**Discussion Activity**<br>• Participants discuss the term patient-oriented research and how they define it<br><br>**Fast Feedback Form**<br>• Participants to rate agreement with statements (strongly agree to strongly disagree)<br>○ *"I understand the history of POR"*<br>○ *"I can describe how POR is different from traditional health research"*	**Post-Workshop Survey**<br>• Participants to rate the workshop sections (excellent to poor)<br>○ *Shared meaning and common language*<br>○ *History and context*<br>○ *Roles of patients in POR and spectrum of engagement*<br>○ *How it works*<br>• Best/most useful part of workshop<br>• Opportunities for improvement<br>• Pacing<br>• Facilitation<br>• Other comments
Articulate why it is beneficial to involve patients in health research.	**Discussion Activity**<br>• Participants share examples of patient engagement success stories	--
Compare patient-reported outcome measures and patient-reported experience measures with measures traditionally used in health research.	**Discussion Activity**<br>• Participants share examples of PROMs	--
B. History and context		
Appreciate the guiding principles that underpin patient engagement in health research: inclusiveness, support, mutual respect and co-building.	--	**Follow-Up Survey**<br>• Participant agreement with guiding principles (yes, always to no, never)<br>○ *"I have observed patient engagement guiding principles being used in health research"*

Learning Outcomes	In-Workshop Evaluation Elements	Post-Workshop Evaluation Elements
Describe examples of ways patients have been involved in patient oriented research.	**Engagement Scenario Activity**<br>Participants are given scenarios and asked to identify the level of participation	--
Appreciate the value of personal stories and how they contribute to a better understanding of the needs, values and preferences of patients.	**Discussion Activity**<br>• Participants discuss the value of patient stories	**Follow Up Survey**<br>• Participants to indicate opportunities to share story (yes or no)<br>○ *"In the past six months, I have had an opportunity to share my story" (describe) OR*<br>○ *"In the past six months, patients have had an opportunity to share their story"*
Assess the unique strengths that patients may bring, not only as patients but through their other personal, educational and professional experiences.	**Small Group Discussion**<br>• Participants share personal backgrounds and how they contribute to research<br><br>**Fast Feedback Form**<br>• Participants to rate agreement with statements (strongly agree to strongly disagree)<br>○ *"I appreciate the value of personal stories and how they contribute to better understanding*	--
C. Roles of patients in POR		
Describe the spectrum of participation as outlined by the International Association of Public Participation (IAP2).	**Ladder of Participation Activity**<br>• Participants put the levels of participation in order<br><br>**Engagement Scenario Activity**<br>• Participants are given scenarios and asked to identify the level of participation	**Follow Up Survey**<br>• Participant to indicate the levels they have been engaged (from patient or researcher perspective)
Describe the various roles that patients can meaningfully and	**Gallery Walk Activity**	**Outcome Indicator**

Learning Outcomes	In-Workshop Evaluation Elements	Post-Workshop Evaluation Elements
actively play in health research, including governance, priority setting, peer review, and other committee work, and the conduct of research itself.	• Participants discuss the research process and the role of patients<br><br>**Fast Feedback Form**<br>• Participants to rate agreement with statements (strongly agree to strongly disagree)<br>○ *"I understand the role of patients in POR and the levels of engagement"*	• To be developed by the evaluation working group
D. How it works		
Outline the practical considerations for engaging patients are partners in health research – e.g. compensation, incentives and rewards, culturally and socially safe environments.	--	**Post-Workshop Survey**<br>• Section rating
Identify the kinds of roles that they are interested in.	--	**Post-Workshop Survey**<br>• Participants to identify roles they are interested in (either being engaged in for patients, or engaging patients in for researchers)<br><br>**Follow Up Survey**<br>• Participants to indicate opportunities to engage in roles<br>○ *In the past six months, I have had an opportunity to get involved in the roles that interest me (describe) OR*<br>○ *"In the past six months, I have had an opportunity to engage patients in roles"*
Identify future learning needs related to those roles.	--	**Post-Workshop Survey**

Learning Outcomes	In-Workshop Evaluation Elements	Post-Workshop Evaluation Elements
		• Participants to identify future learning needs<br><br>**Follow Up Survey**<br>• Participants to identify ongoing learning needs<br>○ *I have identified the following learning needs:*

Evaluation Tools

Fast Feedback Form [paper-copy only]
Distribute this form at the end of the Module 1 session and collect before participants leave.

Fast Feedback!
Please take a few moments to provide some quick feedback on today's session.

In the session today, what perspective were you bringing? (circle one)

Patient – Researcher – Health care professional – Health system policy/decision-maker – Other

1. How did we do in meeting today's learning objectives? Please select one response for each objective.

After today's session:

I understand the history of patient-oriented research.

Strongly Agree – Agree – Disagree – Strongly Disagree

I can describe how POR is different from traditional health research.

Strongly Agree – Agree – Disagree – Strongly Disagree

I understand the role of patients in POR and the levels of engagement.

Strongly Agree – Agree – Disagree – Strongly Disagree

I appreciate the value of personal stories and how they contribute to better understanding.

Strongly Agree – Agree – Disagree – Strongly Disagree

2. What did you find most valuable or helpful about this session?

____

____
____

3. How could this session be improved?

____
____
____

Thank you for your feedback!

Please watch your email – we will also be sending out an online survey to gather additional reflections and comments on today's session.

Post-Workshop Survey

The recommended timeframe for participants to complete this survey is one week after the workshop.

Thank you for participating in the *Module 1: Patient-Oriented Research* session last week. As part of our ongoing improvement efforts, we would appreciate if you could take a few moments to provide some feedback on the session. Your responses are anonymous and will be used only for the purpose of session improvements.

When you participated in the session, what perspective were you bringing? (circle one)

Patient – Researcher – Health care professional – Health system policy/decision-maker – Other

Workshop Sections

1. During the workshop, we covered several sections of content. Please rate each of the workshop sections.

Shared meaning and common language. *Included defining terms such as: patient, engagement, patient-oriented research, and patient-reported outcome measures.*

Excellent - Good - Adequate - Poor - Very Poor

History and context. *Included history of patient engagement, principles of patient and family centred care, patient engagement success stories, partnership with Canadian health research, and Strategy for Patient-Oriented Research (SPOR).*

Excellent - Good - Adequate - Poor - Very Poor

Roles of patients in POR and spectrum of engagement. *Included the research cycle, levels of participation, spectrum of engagement and patient engagement in research.*

Excellent - Good - Adequate - Poor - Very Poor

How it works. *Included practical considerations for patient engagement.*

Excellent - Good - Adequate - Poor - Very Poor

Workshop Format and Delivery

2. Please rate your agreement with the following statements.

The workshop materials were well-prepared.

Strongly Agree - Agree - Disagree - Strongly Disagree

The pace of the workshop was appropriate.

Strongly Agree - Agree - Disagree - Strongly Disagree

The workshop activities were valuable.

Strongly Agree - Agree - Disagree - Strongly Disagree

The workshop facilitation was effective.

Strongly Agree - Agree - Disagree - Strongly Disagree

3. This module is Module 1 in a series. Please indicate which modules you have completed and in which order.

- Module 1 (first – second – third – have not yet taken)
- Module 2 (first – second – third – have not yet taken)
- Module 3 (first – second – third – have not yet taken)

4. This module was delivered as an in-person workshop. Would you have preferred to learn this content in a different way?

   Yes or **No**

   If yes, what other method(s) would you have preferred? (for example, by webinar, online module, etc.)

   ____

5. What will you remember most about the session?

   ____
   ____
   ____

6. What improvements would you suggest?

   ____
   ____
   ____

Patient Engagement in Research

7. **For patients:** During the session we talked about the different roles for patients in research. When you think about getting involved, which roles most interest you? (Check all that apply).

Governance	Priority Setting	Peer Review
Other Committee Work	Research	Other (please describe)

   For researchers: During the session we talked about the different roles for patients in research. When you think about patient involvement in your research, which roles are you currently or are likely to engage them in? (Check all that apply).

Governance	Priority Setting	Peer Review
Other Committee Work	Research	Other (please describe)

8. What other learning needs do you have related to patient-oriented research?

   ____
   ____

9. What goals do you have for your engagement in patient-oriented research?

   ____
   ____

Other Feedback

10. Please share any other comments you have about the workshop.

   ____
   ____
   ____

Thank you for your feedback!

Follow-Up Survey

The recommended timeframe for participants to complete this survey is six months after the workshop.

In the past six months, you participated in the *Module 1: Patient-Oriented Research* session. One of the goals of the workshop is to build capacity for engaging patients in research. Please take a few moments to provide feedback on your participation in patient-oriented research to date.

When you participated in the session, what perspective were you bringing? (circle one)

Patient – Researcher – Health care professional – Health system policy/decision-maker – Other

Engagement in Patient-Oriented Research (POR)

1. **For patients:** After the workshop, you were asked how you wanted to be engaged in POR. In the past six months, have you had an opportunity to be engaged in POR?

   Yes or **No**

   If yes, how have you been engaged in POR? (Check all that apply.)

Governance	Priority Setting	Peer Review
Other Committee Work	Research	Other (please describe)

   If no, what are some of the reasons why you have not been engaged in POR?

   ____

   ____

   For researchers: After the workshop, you were asked how you might engage patients in your research projects. In the past six months, have you had an opportunity to engage patients in POR?

   Yes or **No**

   If yes, how have these patients been engaged in POR? (Check all that apply.)

Governance	Priority Setting	Peer Review
Other Committee Work	Research	Other (please describe)

   If no, what are some of the reasons why you have not engaged patients in POR?

   ____

   ____

Guiding Principles

2. **For patients:** During the workshop, we looked at guiding principles of patient engagement. In thinking of your experiences in health research during the last six months, please select the most appropriate response for the following statements.

   I believe that the research is ***inclusive*** *and includes a diversity of patients' perspectives.*

   Always - Usually - Sometimes - Rarely - Never

   I feel that I have had adequate ***support*** *and flexibility.*

Always - Usually - Sometimes - Rarely - Never

*On the teams that I've worked with, I feel that there is **mutual respect**.*

Always - Usually - Sometimes - Rarely - Never

*I feel that I have had the opportunity to **co-build** by working in partnership from the beginning.*

Always - Usually - Sometimes - Rarely - Never

For researchers: During the workshop, we looked at guiding principles of patient engagement. In thinking of your experiences in health research during the last six months, please select the most appropriate response for the following statements.

*I believe that the research is **inclusive** and includes a diversity of patients' perspectives.*

Always - Usually - Sometimes - Rarely - Never

*I feel that patients have had adequate **support** and flexibility.*

Always - Usually - Sometimes - Rarely - Never

*On the teams that I've worked with, I feel that there is **mutual respect**.*

Always - Usually - Sometimes - Rarely - Never

*In our projects we have had the opportunity to **co-build** by working in partnership from the beginning.*

Always - Usually - Sometimes - Rarely - Never

3. Is there anything you would like to share about your experiences and the guiding principles?

____
____
____

Sharing Stories

4. **For patients:** In the workshop, we talked about the importance of sharing stories. In the past six months, have you had an opportunity to share your story?

Yes or **No**

If yes, Please describe (when, where, how it contributed to the research process).

____
____
____

If no, What are some of the reasons why you have not had an opportunity to share your story?

____
____

____

For researchers: In the workshop, we talked about the importance of sharing stories and. In the past six months, have you had an opportunity to have a patient share their story with you or your research team?

Yes or **No**

If yes, please describe (when, where, how it contributed to the research process).

____
____
____

If no, what are some of the reasons why not?

____
____
____

Ongoing Learning Needs

5. After the workshop, we asked you to identify any future learning needs you might have. In the past six months, have you engaged in other learning opportunities related to POR?

Yes or **No**

If yes, What learning opportunities have you had?

____
____
____

If no, What are some of the reasons you have not engaged in other learning opportunities?

____
____
____

6. What other learning needs do you have related to patient-oriented research?

____
____
____

Other Comments

7. Is there anything else you would like to share about the workshop or your experiences to date with patient-oriented research?

____
____
____

Your insights are valuable for helping us understand how we can better support putting learning into action. Thank you for your feedback!

Facilitator Feedback Survey
The recommended timeframe for completing this survey is no more than one week after the session.

Facilitator Feedback

As part of the pilot testing of the *Module 1: Patient-Oriented Research* session we are looking for feedback from the session facilitators. Please take a few moments to share some insights from your recent session.

1. Please rate your agreement with the following statements:

The workshop materials were well-prepared.

Strongly Agree - Agree - Disagree - Strongly Disagree

The facilitation guide provided the information I needed to deliver the session.

Strongly Agree - Agree - Disagree - Strongly Disagree

The pace of the workshop was appropriate.

Strongly Agree - Agree - Disagree - Strongly Disagree

The session seemed to flow well.

Strongly Agree - Agree - Disagree - Strongly Disagree

2. Were there any concepts or activities that participants seemed to struggle with during the session? If so, please describe.

____
____
____

3. Overall, what do you think is working well with the workshop?

____
____
____

4. What areas for improvement have you identified?

____
____
____

5. Is there anything more you would like to share about your session?

____
____
____

Thank you for your feedback!

Module 2: Fundamentals of Health Research in Canada

Key Evaluation Elements

Learning Outcomes	In-Workshop Evaluation Elements	Post-Workshop Evaluation Elements
A. The Why of Health Research		
Describe the purpose of health research.	**Discussion Activity**<br>• Participants discuss the benefits of health research.<br><br>**Fast Feedback Form**<br>• Participants to rate agreement with statement (strongly agree to strongly disagree)<br>○ *"I can describe the purpose of health research."*	**Post-Workshop Survey**<br>• Participants to rate the workshop sections (excellent to poor)<br>○ *The why of health research*<br>○ *The who of health research*<br>○ *The what of health research*<br>○ *The how of health research*<br>• Best/most useful parts of workshop<br>• Opportunities for improvement<br>• Pacing<br>• Facilitation<br>• Other comments
B. The Who of Health Research		
Describe who typically conducts health research studies and the traditional role of patients as study subjects	**Discussion Activity**<br>• Participants share their ideas of who does research.<br><br>**Fast Feedback Form**<br>• Participants to rate agreement with statement (strongly agree to strongly disagree)<br>○ *"I can describe who conducts health research and the various roles involved (including the roles of patients and*	**Post-Workshop Survey**<br>• See above

Learning Outcomes	In-Workshop Evaluation Elements	Post-Workshop Evaluation Elements
	funders such as CIHR).”	
Describe the role of the Canadian Institutes of Health Research (CIHR) and other health research funders	**Fast Feedback Form**<br>• Participants to rate agreement with statements (strongly agree to strongly disagree)<br>○ *“I can describe who conducts health research and the various roles involved (including the roles of patients and funders such as CIHR).”*	**Post-Workshop Survey**<br>• See above
C. The What of Health Research		
Describe the diversity of health research topics and studies	**Discussion Activity**<br>• Participants identify examples of different research questions for each category.<br><br>**Fast Feedback Form**<br>• Participants to rate agreement with statement (strongly agree to strongly disagree)<br>○ *“I understand the different kinds of health research and can explain the differences between them.”*	**Post-Workshop Survey**<br>• See above
Be aware that there are different research designs and methodologies.	**Crossword Puzzle Activity**<br>• Participants are able to match definitions to terms.	**Post-Workshop Survey**<br>• See above
Define knowledge translation/knowledge exchange	**Crossword Puzzle Activity**<br>• Participants are able to match definitions to terms.	**Post-Workshop Survey**<br>• See above

Learning Outcomes	In-Workshop Evaluation Elements	Post-Workshop Evaluation Elements
Describe the characteristics of a good research question using the FINER acronym (feasible, interesting, novel, ethical and relevant)	**Crossword Puzzle Activity**<br>Participants are able to match definitions to terms.<br><br>**Fast Feedback Form**<br>• Participants to rate agreement with statement (strongly agree to strongly disagree)<br>○ *"I can describe the characteristics of a good research question."*	**Post-Workshop Survey**<br>• See above
D. The How of Health Research		
Describe the stages of a research study	**Exploring the Research Process Activity**<br>• Participants are able to work through the stages of a research study using an example<br><br>**Fast Feedback Form**<br>• Participants to rate agreement with statements (strongly agree to strongly disagree)<br>○ *"I can describe the stages of a research study."*<br>○ *I can describe the difference between evidence and experience."*	**Post-Workshop Survey**<br>• See above
Describe the peer review process	--	**Post-Workshop Survey**<br>• See above
Describe the ethical considerations for health research and how ethical practices are assured	**Crossword Puzzle Activity**<br>• Participants are able to match definitions to terms.	**Post-Workshop Survey**<br>• See above

Evaluation Tools

Fast Feedback Form [paper-copy only]
Distribute this form at the end of the Module 2 session and collect before participants leave.

Fast Feedback!

Please take a few moments to provide some quick feedback on today's session.

In the session today, what perspective were you bringing? (circle one)

Patient – Researcher – Health care professional – Health system policy/decision-maker – Other

1. How did we do in meeting today's learning objectives? Please select one response for each objective.

After today's session:

I can describe the purpose of health research.

Strongly Agree – Agree – Disagree – Strongly Disagree

I can describe who conducts health research and the various roles involved (including the roles of patients and funders such as CIHR).

Strongly Agree – Agree – Disagree – Strongly Disagree

I understand the different kinds of health research and can explain the differences between them.

Strongly Agree – Agree – Disagree – Strongly Disagree

I can describe the stages of a research study.

Strongly Agree – Agree – Disagree – Strongly Disagree

I can describe the characteristics of a good research question.

Strongly Agree – Agree – Disagree – Strongly Disagree

I can describe the difference between evidence and experience.

Strongly Agree – Agree – Disagree – Strongly Disagree

2. What did you find most valuable or helpful about this session?

____

____

3. How could this session be improved?

____

____

Thank you for your feedback!

Please watch your email – we will also be sending out an online survey to gather additional reflections and comments on today's session.

Post-Workshop Survey

The recommended timeframe for participants to complete this survey is one week after the workshop.

Thank you for participating in *Fundamentals of Health Research in Canada* last week. As part of our ongoing improvement efforts, we would appreciate if you could take a few moments to provide some feedback on the session. Your responses are anonymous and will be used only for the purpose of session improvements.

Workshop Sections

1. During the workshop, we covered several sections of content. Please rate each of the workshop sections.

The why of health research. *Included looking at the purpose of health research and how it benefits our health system.*

Excellent - Good - Adequate - Poor - Very Poor

The who of health research. *Included looking at all the people who might be involved in health research (i.e., professors, patients, government) and how that research is funded (such as role of the Canadian Institute for Health Research or CIHR).*

Excellent - Good - Adequate - Poor - Very Poor

The what of health research. *Included looking research categories, research design or methodology, and what makes a good research question.*

Excellent - Good - Adequate - Poor - Very Poor

The how of health research. *Included the stages of research, peer review, and ethics.*

Excellent - Good - Adequate - Poor - Very Poor

Workshop Format and Delivery

2. Please rate your agreement with the following statements.

The workshop materials were well-prepared.

Strongly Agree - Agree - Disagree - Strongly Disagree

The pace of the workshop was appropriate.

Strongly Agree - Agree - Disagree - Strongly Disagree

The workshop activities were valuable.

Strongly Agree - Agree - Disagree - Strongly Disagree

The workshop facilitation was effective.

Strongly Agree - Agree - Disagree - Strongly Disagree

As a result of the workshop, I have a better understanding of health research in general and my role in patient-oriented research.

Strongly Agree - Agree - Disagree - Strongly Disagree

3. What will you remember most about the session?

____
____
____

4. What improvements would you suggest?

____
____
____

Other Feedback

5. Please share any other comments you have about the workshop.

____
____
____

Thank you for your feedback!

Facilitator Feedback Survey
The recommended timeframe for completing this survey is no more than one week after the session.

Facilitator Feedback

As part of the pilot testing of the *Fundamentals of Health Research in Canada Workshop* we are looking for feedback from the session facilitators. Please take a few moments to share some insights from your recent session.

1. Please rate your agreement with the following statements:

   The workshop materials were well-prepared.

   Strongly Agree - Agree - Disagree - Strongly Disagree

   The facilitation guide provided the information I needed to deliver the session.

   Strongly Agree - Agree - Disagree - Strongly Disagree

   The pace of the workshop was appropriate.

   Strongly Agree - Agree - Disagree - Strongly Disagree

   The session seemed to flow well.

   Strongly Agree - Agree - Disagree - Strongly Disagree

2. Were there any concepts or activities that participants seemed to struggle with during the session? If so, please describe.
   ____
   ____
   ____

3. Overall, what do you think is working well with the workshop?
   ____
   ____
   ____

4. What areas for improvement have you identified?
   ____
   ____
   ____

5. Is there anything more you would like to share about your session?
   ____
   ____
   ____

Thank you for your feedback!

Module 3: Building Partnerships and Consolidating Teams

Key Evaluation Elements

<table>
<tr><th>Learning Outcomes</th><th>In-Workshop
Evaluation Elements</th><th>Post-Workshop
Evaluation Elements</th></tr>
<tr><td colspan="3">A. Understanding team development</td></tr>
<tr><td>Explain Tuckman’s stages of team development (forming, storming, norming, performing) and;

Develop strategies to work through each of the stages.</td><td>Learning Activity – Diversity in the Room
• Participants discuss common goals or purpose.

Learning Activity – About to Storm
• Participants discuss behaviors and strategies for the storming stage.

Learning Activity – Case Study Vignette
• Participants discuss stages of team development based on case study.

Learning Activity – Norming
• Participants discuss personal examples of teams that have been in “norming” stage.
Learning Activity – Performing
• Participants discuss own experiences with performing.

Fast Feedback Form
• Participants to rate agreement with statement (strongly agree to strongly disagree)
○ “I can explain the stages of team development.”
○ “I can identify strategies for working through each of the stages.”</td><td>Post-Workshop Survey
• Participants to rate the workshop sections (excellent to poor)
○ Learning how to do research as a team
○ Approaches and tools for stages of team development
○ Building mutually beneficial partnerships
• Best/most useful parts of workshop
• Opportunities for improvement
• Pacing
• Facilitation
• Other comments

Follow Up Survey
• Participant agreement with team development strategies (yes, always to no, never)
○ “In the teams that I have been involved with, we have been able to recognize and work through the stages of team development”</td></tr>
</table>

Learning Outcomes	In-Workshop Evaluation Elements	Post-Workshop Evaluation Elements
B. Review and integrate concepts from previous modules		
Be able to put the guiding principles for patient-oriented research into action.	**Fast Feedback Form**<br>• Participants to rate agreement with statement (strongly agree to strongly disagree)<br>○ *"I understand how the guiding principles for patient-oriented research can be put into action."*	**Post-Workshop Survey**<br>• See above<br><br>**Follow Up Survey**<br>• Participant agreement with guiding principles (yes, always to no, never)<br>○ *"In the teams that I have been involved with, I feel that the guiding principles are being put into action"*
C. Approaches and tools for effective teamwork		
Understand how:<br>• How roles and responsibilities are viewed differently through the lenses of culture, class, gender, etc.<br>• How people's confidence and ability to use their voice can be undermined.<br>• How our current system can impede work in partnership.<br>• How to leverage experience-based knowledge within a team.<br>• How learning and leadership styles have to be taken into account to adjust partnership dynamics.	**Fast Feedback Form**<br>• Participants to rate agreement with statement (strongly agree to strongly disagree)<br>○ *I recognize the different roles that team members can play and their importance.*<br>○ *I've identified the roles that suit me and those that don't*<br>○ *I know what it takes to ensure team members feel comfortable to contribute ideas*<br>○ *I can see how leadership within a team can be shared according to each members' expertise*	--
Learn about different ways to support team work.	**Fast Feedback Form**<br>• Participants to rate agreement with statement (strongly agree to strongly disagree)	**Follow Up Survey**<br>• Participant agreement with the statements pertaining to experience of being a member of a team after

Learning Outcomes	In-Workshop Evaluation Elements	Post-Workshop Evaluation Elements
	○ *"I can recognize when a team is vulnerable and might fall apart"*<br>○ *"I can describe the team member behaviours that can threaten team development"*	module 3 (strongly agree to strongly disagree)<br>○ *"In the teams that I have been involved with, I am confident that I was able to act appropriately to support team development"*<br>○ *In the teams that I have been involved with, I was able to identify the behaviors by other team members that threatened our team"*
Learn about effective decision-making models/processes for partnership.	**Learning Activity – Types of Decisions**<br>• Participants discuss own experiences with decision making approaches.<br><br>**Fast Feedback Form**<br>• Participants to rate agreement with statement (strongly agree to strongly disagree)<br>○ *"I can describe effective decision-making processes"*	**Post-Workshop Survey**<br>• See above<br><br>**Follow Up Survey**<br>• Participant agreement with guiding principles (yes, always to no, never)<br>○ *"In the teams that I have been involved with, we have used effective decision making models to guide our work"*
D. Building partnerships		
Frame and plan team actions with the CEPPP's 6-stages co-building model.	**Learning Activity – Discussion on Partnership (Slide 7)**<br>• Participants discuss definitions of the term partnership.<br><br>**Learning Activity – CEPPP Model Questions**<br>• Teams work together to answer questions based on the CEPPP model.<br><br>**Fast Feedback Form**<br>• Participants to rate agreement with statement	**Post-Workshop Survey**<br>• See above<br><br>**Follow Up Survey**<br>• Participant agreement with defining role (yes, always to no, never)<br>○ *"In the teams that I have been involved with, we have been applying the CEPPP model to co-build our team"*

Learning Outcomes	In-Workshop Evaluation Elements	Post-Workshop Evaluation Elements
	(strongly agree to strongly disagree)<br>○ *"I can apply the CEPPP model to co-building our team"*	

Evaluation Tools

Fast Feedback Form [paper-copy only]
Distribute this form at the end of the Module 3 session and collect before participants leave.

Fast Feedback!
Please take a few moments to provide some quick feedback on today's session.

In the session today, what perspective were you bringing? (circle one)

Patient – Researcher – Health care professional – Health system policy/decision-maker – Other

How did we do in meeting today's learning objectives? Please select one response for each objective.

1. *After today's session:*

I can explain the stages of team development.

Strongly Agree – Agree – Disagree – Strongly Disagree

I can identify strategies for working through each of these stages.

Strongly Agree – Agree – Disagree – Strongly Disagree

I recognize the different roles that team members can play and their importance.

Strongly Agree – Agree – Disagree – Strongly Disagree

I've identified the roles that suit me and those that don't.

Strongly Agree – Agree – Disagree – Strongly Disagree

I know what it takes to ensure team members feel comfortable to contribute ideas.

Strongly Agree – Agree – Disagree – Strongly Disagree

I can see how leadership within a team can be shared according to each members' expertise.

Strongly Agree – Agree – Disagree – Strongly Disagree

I can recognize when a team is vulnerable and might fall apart.

Strongly Agree – Agree – Disagree – Strongly Disagree

I can describe the team member behaviours that can threaten team development.

Strongly Agree – Agree – Disagree – Strongly Disagree

I understand how the guiding principles for patient-oriented research can be put into action.

Strongly Agree – Agree – Disagree – Strongly Disagree

I can describe effective decision-making processes.

Strongly Agree – Agree – Disagree – Strongly Disagree

I can apply the CEPPP model to co-building our team.

Strongly Agree – Agree – Disagree – Strongly Disagree

2. What did you find most valuable or helpful about this session?

____
____
____
____
____

3. How could this session be improved?

____
____
____
____
____

Thank you for your feedback!

Please watch your email – we will also be sending out an online survey to gather additional reflections and comments on today's session.

Post-Workshop Survey

The recommended timeframe for participants to complete this survey is one week after the workshop.

Thank you for participating in the *Module 3: Building Partnerships and Consolidating Teams Workshop* last week. As part of our ongoing improvement efforts, we would appreciate if you could take a few moments to provide some feedback on the session. Your responses are anonymous and will be used only for the purpose of session improvements.

Workshop Sections

1. During the workshop, we covered several sections of content. Please rate each of the workshop sections.

    Learning to do research as a team. *Looked at guiding principles for patient-oriented research.*

    Excellent - Good - Adequate - Poor - Very Poor

    Approaches and tools for stages of team development. *Included looking at stages of team development and strategies for working through the stages. Also looked at effective decision-making.*

    Excellent - Good - Adequate - Poor - Very Poor

    Building partnerships. *Included learning about the CEPPP model and applying it to team.*

    Excellent - Good - Adequate - Poor - Very Poor

Workshop Format and Delivery

2. Please rate your agreement with the following statements.

    The workshop materials were well-prepared.

    Strongly Agree - Agree - Disagree - Strongly Disagree

    The pace of the workshop was appropriate.

    Strongly Agree - Agree - Disagree - Strongly Disagree

    The workshop activities were valuable.

    Strongly Agree - Agree - Disagree - Strongly Disagree

    The workshop facilitation was effective.

    Strongly Agree - Agree - Disagree - Strongly Disagree

    As a result of the workshop, I have a better understanding of effective team work in health research.

    Strongly Agree - Agree - Disagree - Strongly Disagree

3. Did you attend this workshop with your research team?

   Yes or **No**

   If no, what were the reasons why you did not attend as a team?

   ____
   ____
   ____

4. This module is Module 3 in a series. Please indicate which modules you have completed and in which order.
   - Module 1 (first – second – third – have not yet taken)
   - Module 2 (first – second – third – have not yet taken)
   - Module 3 (first – second – third – have not yet taken)

5. This module was delivered as an in-person workshop. Would you have preferred to learn this content in a different way?

   Yes or **No**

   If yes, what other method(s) would you have preferred? (for example, by webinar, online module, etc.)

   ____
   ____

6. What will you remember most about the session?

   ____
   ____
   ____

7. What improvements would you suggest?

   ____
   ____
   ____
   ____

Other Feedback

8. Please share any other comments you have about the workshop.

   ____
   ____
   ____
   ____

Thank you for your feedback!

Follow-Up Survey

Time-frame for follow-up survey is recommended six months after session.

In the past six months, you participated in the *Module 3: Building Partnerships and Consolidating Teams Workshop.* One of the goals of the workshop is to build capacity for effective teamwork. Please take a few moments to provide feedback on your experience as a team member in a patient-oriented research project.

1. Have you been involved as part of a team on a health care research project?

   Yes or **No**

   If no, what were some of the reasons for not being involved and/or barriers to being part of a team?

   ____
   ____
   ____
   ____

Learning to do Research as a Team

2. During the workshop, we looked doing research as a team. In thinking of your experiences in health research during the last six months, please select the most appropriate response for the following statement.

   In the teams that I have been involved with, I feel that the guiding principles are being put into action.

   Always - Usually - Sometimes - Rarely - Never

3. Is there anything you would like to share about your experiences with team research?

   ____
   ____
   ____
   ____

Approaches and Tools for Effective Teams

4. During the workshop, we looked at approaches and tools for effective teamwork. In thinking of your experiences in health research during the last six months, please select the most appropriate response for the following statements.

   In the teams that I have been involved with, we have been able to recognize and work through the stages of team development.

   Always - Usually - Sometimes - Rarely - Never

   In the teams that I have been involved with, we have used effective decision making models to guide our work.

   Always - Usually - Sometimes - Rarely - Never

In the teams that I have been involved with, I am confident that I was able to act appropriately to support team development.

Always - Usually - Sometimes - Rarely - Never

In the teams that I have been involved with, I was able to identify the behaviors by other team members that threatened our team.

Always - Usually - Sometimes - Rarely - Never

5. Is there anything you would like to share about the effectiveness of your team experience?

____
____
____
____

Building Partnerships

6. During the workshop, we looked at using the CEPPP approach for co-building the team. In thinking of your experiences in health research during the last six months, please select the most appropriate response for the following statement.

In the teams that I have been involved with, we have used the CEPPP model to co-build our team.

Always - Usually - Sometimes - Rarely - Never

7. Is there anything you would like to share about building the team?

____
____
____
____

Other Comments

8. Is there anything else you would like to share about the workshop or your experiences to date with patient-oriented research?

____
____
____
____

Your insights are valuable for helping us understand how we can better support putting learning into action. Thank you for your feedback!

Facilitator Feedback Survey

The recommended timeframe for completing this survey is no more than one week after the session.

Facilitator Feedback

As part of the pilot testing of the *Module 3: Building Partnerships and Consolidating Teams* session we are looking for feedback from the session facilitators. Please take a few moments to share some insights from your recent session.

1. Please rate your agreement with the following statements:

   The workshop materials were well-prepared.

   Strongly Agree - Agree - Disagree - Strongly Disagree

   The facilitation guide provided the information I needed to deliver the session.

   Strongly Agree - Agree - Disagree - Strongly Disagree

   The pace of the workshop was appropriate.

   Strongly Agree - Agree - Disagree - Strongly Disagree

   The session seemed to flow well.

   Strongly Agree - Agree - Disagree - Strongly Disagree

2. Were there any concepts or activities that participants seemed to struggle with during the session? If so, please describe.

   ____
   ____
   ____

3. Overall, what do you think is working well with the workshop?

   ____
   ____
   ____

4. What areas for improvement have you identified?

   ____
   ____
   ____

5. Is there anything more you would like to share about your session?

   ____
   ____
   ____

Thank you for your feedback!
